# Elevated miR-34a induced by lipotoxicity and inflammation mediates pathophysiological communication between hepatocytes and hepatic stellate cells in liver fibrosis

**DOI:** 10.1016/j.gendis.2025.101648

**Published:** 2025-04-18

**Authors:** Qihua Duan, Ruixiang Hu, Yan Chen, Henry Wade, Szczepan Kaluzny, Bingrui Zhang, Rongxue Wu, Guangnan Liu, Cunchuan Wang, Edward N. Harris, Qiaozhu Su

**Affiliations:** a Institute for Global Food Security, School of Biological Sciences, Queen's University Belfast, Belfast BT9 5DL, United Kingdom; b Department of Gastrointestinal Surgery, First Affiliated Hospital of Jinan University, Guangzhou, Guangdong 510630, China; c Second Affiliated Hospital of Guangxi Medical University, Guangxi 530007, China; d Department of Medicine, Section of Cardiology, University of Chicago, Chicago 60637, USA; e Department of Biochemistry, University of Nebraska-Lincoln, Lincoln, NE 68588, USA

**Keywords:** Cell–cell communication, Hepatic stellate cells, Liver fibrosis, MASLD, microRNA-34a

## Abstract

Increased mortality in patients with metabolic dysfunction-associated steatotic liver disease (MASLD) imposes an urgent need to elucidate the pathogenesis of MASLD so that novel therapeutic strategies may be identified. Here, we delineate the mechanism of microRNA-34a-5p (miR-34a) in the progressive liver injury of MASLD and liver fibrosis. Specifically, liver tissue from patients with obesity-associated hepatic steatosis, metabolic dysfunction-associated steatohepatitis (MASH), and fibrosis, as well as liver tissues from a human MASLD-like mouse model, were utilized for this study. We found that lipotoxicity resulting from obesity or saturated free fatty acid treatment induced miR-34a expression in human liver tissue or mouse hepatocytes, which was accompanied by dysregulation of lipoprotein metabolism, activation of inflammation, and ballooning degeneration of hepatocytes. Moreover, increased cellular miR-34a induced by treatment with saturated fat, palmitic acid, or transfection of miR-34a mimic was released from injured hepatocytes into the conditional cell culture media, which mediated pathological communications between hepatocytes and hepatic stellate cells, activated pro-fibrogenic signaling in hepatic stellate cells, and induced extracellular matrix remodeling. These phenotypes were recapitulated in a human MASLD-like mouse model in which MASLD and liver fibrosis were induced via streptozotocin treatment and high-fat feeding. Elevated expression of miR-34a was found in mouse liver tissues, which conveyed the progressive liver injury from steatosis to MASH and liver fibrosis. Our findings demonstrate that elevated miR-34a induced by lipotoxicity and metabolic inflammation are key driving factors in the progressive liver injury from simple steatosis to MASH and liver fibrosis.

## Introduction

Metabolic dysfunction-associated steatotic liver disease (MASLD), previously referred to as non-alcoholic fatty liver disease (NAFLD), is a multifactorial disease that encompasses a spectrum of pathological conditions, ranging from simple steatosis, metabolic dysfunction-associated steatohepatitis (MASH), and fibrosis/cirrhosis, which can further progress to hepatocellular carcinoma and liver failure.^1^^,^^2^ MASLD is a common complication of obesity, which imposes major challenges on global health. 70%–80% of obese individuals have hepatic steatosis, 15%–30% develop MASH, and one-third of patients progress to liver fibrosis within 4–5 years.^2^, ^3^, ^4^ Initially, the “two-hit” hypothesis was proposed to be the mechanism for MASLD development^5^: the “first hit” is usually the accumulation of lipids, including triglycerides, free fatty acids, and cholesterol in hepatocytes, which induces simple steatosis and increases vulnerability of the liver to injury from the “second hit”. Lipotoxicity, mitochondrial dysfunction, cellular stress, and inflammation are the “second hit” that drives liver damage progressing from simple steatosis to MASH.^6^ Recent research further evolved the “two-hit” hypothesis into the “multiple-hit” hypothesis, which proposes that deleterious factors from insulin resistance, inflammation, dietary factors, lipopolysaccharide generated by the gut microbiota, and genetic or epigenetic disorders are the “multiple hits” driving the development of MASH to fibrosis.^7^^,^^8^ Fibrosis is a key factor for liver disease prognosis and a risk factor of hepatocellular carcinoma. While scientific discoveries within the last decade have transformed our understanding of the mechanisms of liver fibrosis by demonstrating that removal or elimination of the causative factors (*e.g.*, obesity) can prevent or slow down disease progression to liver fibrosis, the reversal action often occurs too slowly or too infrequently to avoid the life-threatening complications, particularly in advanced fibrosis. Thus, there is a huge unmet medical need for anti-fibrotic therapies to prevent liver disease progression and hepatocellular carcinoma development.

Hepatocytes are the dominant hepatic cell type that perform most liver functions, including energy metabolism, detoxification, and nutrient storage in the liver. The cAMP-responsive element binding protein H (CREBH) is a transcription factor exclusively expressed in the liver and intestine, which regulates metabolic pathways and inflammatory responses, such as lipid and lipoprotein metabolism, fatty acid β-oxidation, gluconeogenesis, acute-phage response gene expression, and MASH development.^9^, ^10^, ^11^, ^12^ The pathological progression of simple steatosis to liver fibrosis is associated with a series of liver injuries resulting from lipotoxicity, cellular stress, inflammation, hepatocyte ballooning degeneration, and secretion of fibrogenic mediators, *i.e.*, transforming growth factors (TGFs), from damaged hepatocytes.^2^ These adverse mediators communicate with nearby hepatocytes and other liver cells, namely hepatic stellate cells (HSCs), Kupffer cells, and liver sinusoidal endothelial cells, through complex processes, leading to the development of liver fibrosis.^6^^,^^13^ HSCs are located in the hepatic perisinusoidal space of Disse, are proximate to hepatocytes, and exist in a quiescent state.^14^ In response to liver injury, quiescent HSCs trans-differentiate into myofibroblast-like cells (activated HSCs) which express alpha-smooth muscle actin (α-SMA) and a variety of pro-fibrogenic cytokines and proteins, including TGFβ1/2, cellular communication network 2 (CCN2), platelet derived growth factor, fibroblast growth factor, and tissue inhibitors of metalloproteinases that promote remodelling of extracellular matrix and contribute to liver fibrosis and cirrhosis.^2^ Despite HSCs playing a pivotal role in liver fibrosis, emerging evidence demonstrates that injured hepatocytes are the dominant cell type managing the progression of liver fibrosis. Lipotoxicity and endoplasmic reticulum (ER) stress can augment hepatocyte injury. Furthermore, redox imbalance, activation of toll-like receptors (TLRs), apoptosis, and hedgehog signalling can contribute to the development of liver fibrosis.^2^ Thus, therapeutic strategies of inhibiting hepatocyte apoptosis and inactivating HSCs are the currently accepted resolution for liver fibrosis.

MicroRNAs (miRNAs) are endogenous small non-coding RNAs that participate in epigenetic regulation by binding to the seed sequences at the 3′- or 5′-untranslated regions of targeted mRNAs, resulting in decay or translational repression of the targeted mRNAs. miRNAs have been demonstrated to be involved in various metabolic processes in the liver that are associated with MASLD, MASH, and liver fibrosis, *e.g.*, lipid metabolism, inflammation, and cell regeneration.^15^^,^^16^ For instance, miR-103 was up-regulated in the serum of patients with MASLD compared with healthy controls, which was associated with up-regulated adipogenesis in hepatocytes, causing ectopic accumulation of lipids in the liver.^17^ Increased miR-378a3p was reported to induce hepatic metabolic inflammation and insulin resistance in animal models and patients with MASH.^18^^,^^19^ miRNAs (*e.g.*, miR-130, miR-378a) also interacted with long-non-coding RNAs (*e.g.*, H19, SNHG7) and are involved in MASLD and liver fibrosis.^20^^,^^21^ Moreover, the down-regulation of miR-122 or miR-29c was observed in the livers of patients with MASH compared with patients with steatosis or healthy controls.^22^, ^23^, ^24^ These miRNAs targeted genes involved in different metabolic pathways associated with the development of MASLD and fibrosis. However, the underlying mechanisms of miRNAs involved in obesity-associated liver fibrosis remain poorly understood.

In this study, we uncovered a novel mechanism of miR-34a in the pathogenesis of MASLD and liver fibrosis. Specifically, increased miR-34a expression was found in human patients with obesity associated steatosis, MASH, and liver fibrosis, and a MASLD-like mouse model. Lipotoxicity induced by saturated free fatty acids, palmitic acid (PA) treatment, and a high-fat diet induced miR-34a expression, which mediated liver injury via activation of ER stress, metabolic inflammation, hepatocyte ballooning degeneration, sonic hedgehog (SHH), and pro-fibrogenic signaling. Moreover, secreted miR-34a released from injured hepatocytes activated HSCs and induced the remodeling of the extracellular matrix, contributing to the progression of MASLD to MASH and fibrosis.

## Materials and methods

### Human tissue samples

Consent was obtained from each patient included in the study, and the study protocol conformed to the ethical guidelines of the 1975 Declaration of Helsinki as reflected in a priori approval by the institution's human research committee. Clinical samples used in this study were approved by the Ethics Committee of the First Affiliated Hospital of Jinan University in Guangzhou, China (KY-2021-102).

### Animal study

Pathogen-free pregnant mice were purchased from The Jackson Laboratory. All animal experiments were approved by the University of Nebraska–Lincoln Institutional Animal Care and Use Committee (Protocol #2200 at UNL) and were performed according to the NIH guidelines (guide for the care and use of laboratory animals). Animals were housed on alternating 12-h/12-h light/dark cycles with free access to food and water. A human MASLD-like model was established as described before.^25^ Neonatal 2-day-old male mice were subcutaneously injected with a dose of streptozotocin (200 μg/mouse) to destroy pancreatic β-cells, leading to a deficiency of insulin secretion. The streptozotocin-treated mice were then randomly divided into two groups (*n* = 8/group) and subjected to a control chow diet (BioServ #F4031) or high-fat diet (BioServ #F3282) at day 22 (when mice were weaning). These mice are equivalent to the STAM™ mouse model developed by Fugii et al at SMC Laboratories. The livers of the STAM model mice and control mice were collected at 6-, 8-, and 12-weeks of age after being fasted for 12 h and then were frozen in liquid nitrogen and stored at −80 °C before further analysis. This study used the ARRIVE reporting guidelines.^26^

### Primary hepatocytes

Primary hepatocytes were isolated according to the protocol provided by Cabral et al.^27^ Briefly, anesthetized mice were cannulated via the portal vein, and the liver was infused with 0.5 mg/mL type-1 collagenase (Worthington Biochemical) for 12 min at a flow rate of 6 mL/min. The amorphous liver was then physically dissociated in saline, and cells were filtered through 100 μm and 40 μm mesh filters. Hepatocytes were purified by centrifugation at 50 *g* for 3 min, washed with saline, and repeated twice. The effluent contained nonparenchymal cells and dead hepatocytes.

### Histological analysis

Tissue extracted from humans and mice was immediately soaked in formalin (phosphate-buffered saline containing 10% formaldehyde) for 24 h and then processed for hematoxylin–eosin staining, and images were collected with a Nikon light microscope at 40× magnification.

### Hepatocyte ballooning assay and imaging

3 × 10^4^ mouse hepatocytes (AML12 cells) were seeded onto slides in media containing 10% fetal bovine serum. Upon reaching 70% confluence, cells were transfected scramble miRNA mimics (30 nM, Qiagen, GeneGlobe ID - YM00479902) or miR-34a mimic (30 nM, Qiagen, GeneGlobe ID - YM00473212) using Lipofectamine 3000 (Thermofisher, L3000008)/Opti-MEM (Gibco, 31985062). 48 h post transfection, culture media was removed, and cells were fixed with 4% paraformaldehyde (VWR, 158127) and stained with hematoxylin and eosin for 3 min and 30 s, respectively. Slides were then mounted by Fluoromount-G (Invitrogen, E133739) and covered with glass coverslip. Stained cells were captured by an OLYMPUS CX41 microscope at 40× magnification.

### Wound healing assay

1 × 10^5^ HSCs were seeded into a 12-well plate in media containing 10% fetal bovine serum. Upon reaching 100% confluency, the media was removed. The confluent HSC-T6 (HSC) cell monolayer was then scratched using a sterile 2 μL pipette tip (0-h timepoint). After the cell debris was removed, conditioned media prepared from AML12 cells transfected with scramble miRNA mimics or miR-34a were added into the scratched HSCs and incubated for up to 30 h. Images were captured using an inverted light microscope at 0-, 6-, 24- and 30-h timepoints. The wound area was quantified using ImageJ, and the healing rate was determined by calculating the ratio (%) of wounded area at each time point relative to the wounded area at 0 h.

### Statistical analysis

The Anderson-Darling test on the residuals was used to test for normality, and Levene's test was used to test for equal variances. The two-tailed student's *t*-test was used for statistical analyses of two-group comparisons. All results were presented as means ± standard deviation. Asterisks (∗ or ∗∗) indicate statistically significant differences of *p* < 0.05 or < 0.01, respectively, versus controls.

## Results

### Elevated miR-34a induced by lipotoxicity activated inflammatory signaling in hepatocytes

**Figure 1:**
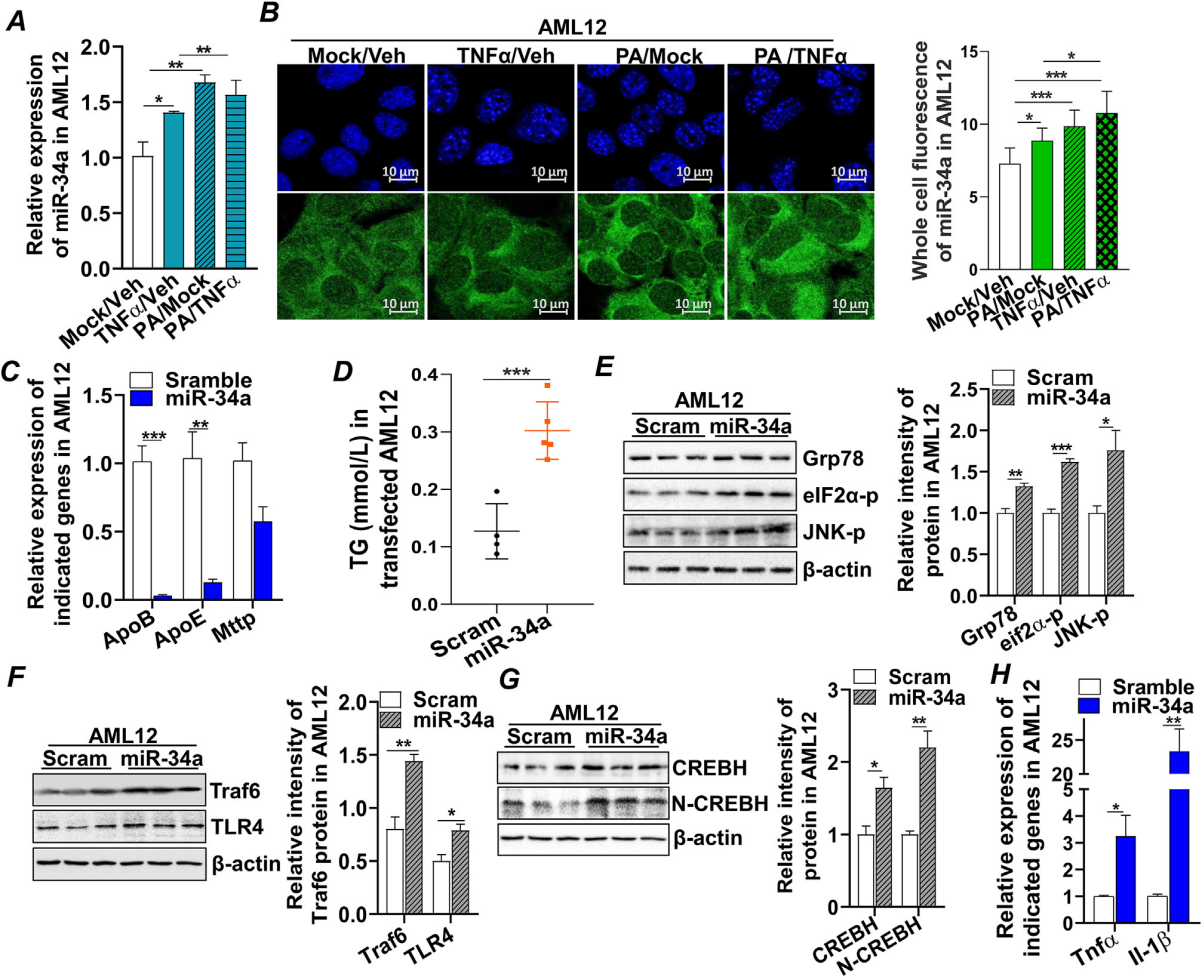
Elevated miR-34a induced by lipotoxicity-activated inflammatory signaling in hepatocytes. **(A)** AML12 cells were treated with Mock (0.5% BSA)/Veh (H_2_O), PA (0.25 mM)/Veh (H_2_O), or TNFα (20 ng/mL)/Mock (0.5% BSA) for 18 h mRNA expression of miR-34a was detected with qRT-PCR. **(B)** Image of miR-34a fluorescent *in situ* hybridization (FISH) signals in AML12 cells treated with Mock (0.5% BSA)/Veh (H_2_O), PA (0.25 mM)/Veh (H_2_O), or TNFα (20 ng/mL)/Mock (0.5% BSA) for 18 h. Scale bar, 10 μm. **(C)** mRNA expression of lipoproteins *ApoB*, *ApoE*, and *Mttp* by qRT-PCR in AML12 cells transfected with Scram (control) or miR-34a mimics for 48 h. **(D)** TG in AML12 cells transfected with Scram or miR-34a mimics for 48 h. **(E)** Immunoblotting analysis of GRP78, eIF2α-p, JNK-p, and loading control β-actin protein in the AML12 cells transfected with Scram or miR-34a mimics for 48 h. **(F)** mRNA expression of *Tnfα* and *Il-1β* by qRT-PCR in AML12 cells transfected with Scram (control) or miR-34a mimics for 48 h. **(G, H)** Immunoblotting analysis of TRAF6, TLR4, CREBH, N-CREBH, and loading control β-actin protein in the AML12 cells transfected with Scram or miR-34a mimics for 48 h. Results represent mean ± standard deviation. The two-tailed student's *t*-test was used for statistical analyses of two-group comparisons. ∗*p* < 0.05, ∗∗*p* < 0.01, and ∗∗∗*p* < 0.001 versus controls. Veh, vehicle; Scram, scramble miRNA; BSA, bovine serum albumin; PA, palmitic acid; TNFα, tumor necrosis factor alpha; qRT-PCR, real-time quantitative reverse transcription PCR; ApoB, apolipoprotein B; ApoE, apolipoprotein E; Mttp, microsomal triglyceride transfer protein; TG, triglyceride; GRP78, glucose regulatory protein 78; eIF2α-p, phosphor-eukaryotic initiation factor-2α; JNK-p, phosphor-c-Jun N-terminal kinase; Il-1β, interleukin-1β; TRAF6, tumor necrosis factor receptor-associated factor 6; TLR4, toll-like receptor 4; CREBH, cAMP-responsive element binding protein H; N-CREBH, N-terminal CREBH.

Lipotoxicity and the subsequent inflammation are two crucial “hits” in the initiation and progression of MASLD. To mimic this pathological condition, the mouse hepatocyte cell line (AML12) was treated with free saturated fatty acids, PA, and/or inflammatory cytokine tumor necrosis factor-alpha (TNFα, 20 ng/mL). We found that expression of miR-34a was significantly up-regulated by PA or TNFα treatment (Fig. 1A). To confirm this observation, a miR-34a fluorescent *in situ* hybridization assay was conducted on AML12 with the same treatment and we found that cellular miR-34a was primarily localized in the cytoplasm, and PA and/or TNFα treatments up-regulated miR-34a expression (Fig. 1B). To determine the impact of miR-34a up-regulation on hepatocytes, AML12 were transfected with synthetic miR-34a mimics and found that increased miR-34a enhanced expression of genes involved in hepatic *de novo* lipogenesis, such as *Srebp*-1c, *Fasn*, *Acc*, and *Scd1*, which was associated with the increased triglyceride contents in the hepatocytes (Fig. S1A, D). Expression of S*rebp*-2 mRNA, a transcription factor mainly involved in cholesterol synthesis, was not changed by miR-34a transfection, which was consistently reflected in the cholesterol content in the transfected AML12 cells (Fig. S1A, B). On the other hand, increased miR-34a inhibited expression of genes that regulated hepatic very low-density lipoprotein (VLDL) assembly, including apolipoprotein B (*ApoB*), apolipoprotein E (*ApoE*), and microsomal triglyceride transfer protein *(Mttp)* (Fig. 1C), contributing to the accumulation of lipids in AML12 (Fig. 1D). Lipotoxicity from the accumulated lipids induced ER stress in the miR-34a-transfected cells, indicated by the activation of ER stress markers, glucose regulatory protein 78 (GRP78), phosphor-eukaryotic initiation factor-2α (eIF2α-p), and phosphor-c-Jun N-terminal kinase (JNK-p), compared with the mock controls (Fig. 1E). miR-34a further activated TLR4 and the tumor necrosis factor receptor-associated factor 6 (TRAF6) signaling pathway (Fig. 1F; Fig. S1C), a pathway that was previously reported to enhance cleavage and activation of CREBH, a hepatic transcription factor involved in acute phase response and inflammatory signaling.^28^^,^^29^ Indeed, immunoblotting analysis showed that expression of CREBH precursor (full-length) and active CREBH (N-terminal CREBH) were up-regulated upon miR-34a transfection, which was accompanied by increased expression of inflammatory cytokines *Tnf*α and interleukin-1β (*Il-1β*) (Fig. 1G, H). Collectively, these data demonstrated that lipotoxicity and metabolic inflammation could induce miR-34a expression, which in turn dysregulated hepatic lipid and VLDL metabolism, leading to lipotoxicity and metabolic inflammation that contribute to the development of steatosis in hepatocytes.

### miR-34a overexpression induced hepatocyte ballooning degeneration and fibrogenic status

**Figure 2:**
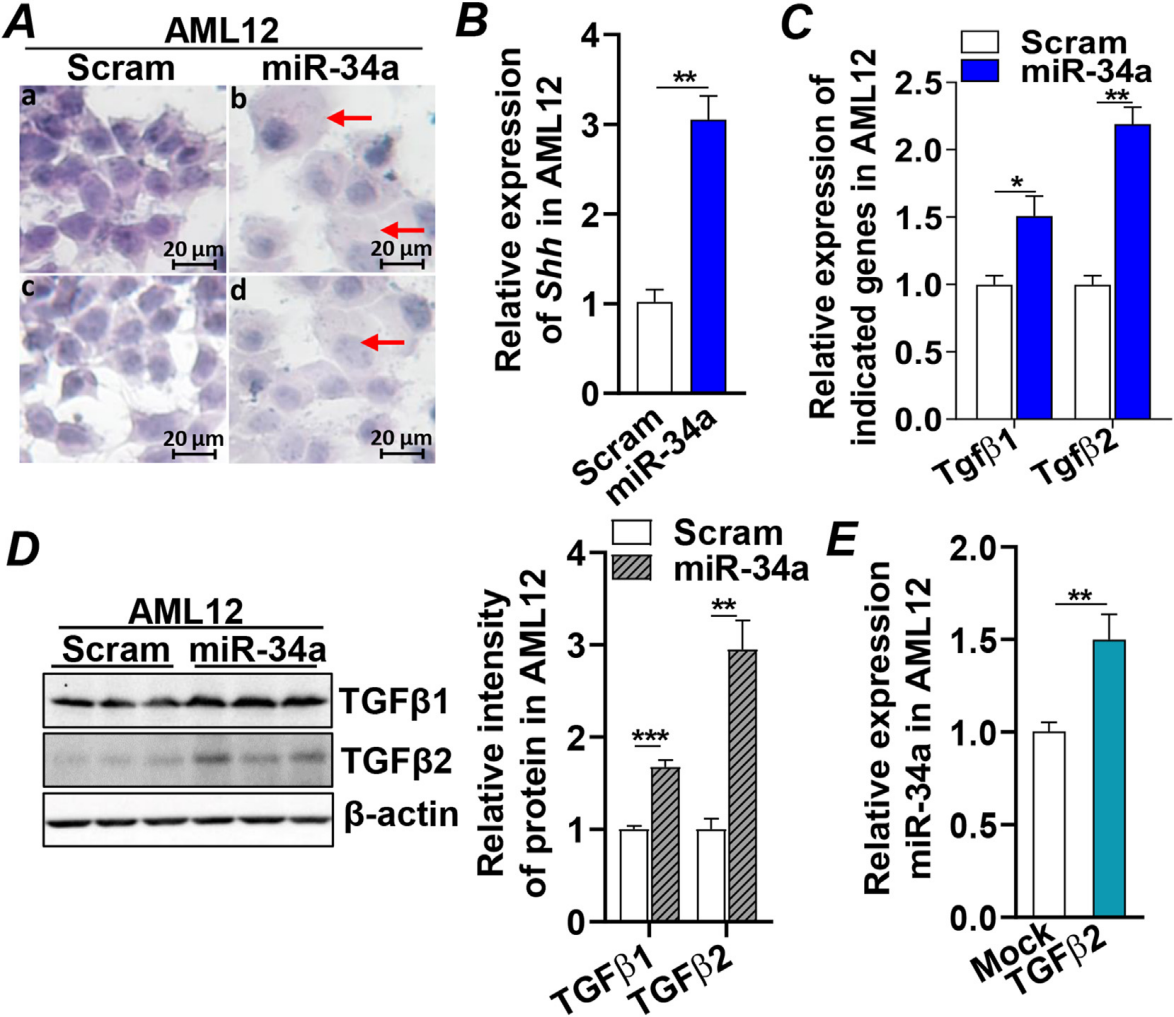
Increased miR-34a induced hepatocyte ballooning degeneration and fibrogenic status. AML12 cells were transfected with Scram (control) or miR-34a mimics for 48 h and subjected to the following analysis (A–D). **(A)** Hematoxylin–eosin staining and microscope imaging of the transfected AML12 cells. The arrows mark the ballooned AML12 cells. Scale bar, 20 μm. **(B)** mRNA expression of *Shh* by qRT-PCR. **(C)** qRT-PCR analysis of *Tgfβ1* and *Tgfβ2* mRNAs. **(D)** Immunoblotting analysis of TGFβ1 and TGFβ2 and loading control β-actin proteins. **(E)** mRNA expression of miR-34a by qRT-PCR in AML12 cells treated with Veh (H_2_O) or Tgfβ2 (2 mg/mL) for 48 h. For cell treatment, two independent experiments were performed in triplicate. Results represent mean ± standard deviation. The two-tailed student's *t*-test was used for statistical analyses of two-group comparisons. ∗*p* < 0.05 and ∗∗*p* < 0.01 versus controls. qRT-PCR, real-time quantitative reverse transcription PCR; Shh, sonic hedgehog; TGF-β1/2, transforming growth factor-beta 1/2; Veh, vehicle; Scram, scramble miRNA.

Hepatocyte ballooning degeneration is a pathological hallmark of MASH and is associated with the development of liver fibrosis. Transfection of miR-34a in AML12 for 48 h induced hepatocyte ballooning degeneration, indicated by the presence of enlarged, swollen, and rounded hepatocytes (Fig. 2A). Consistently, expression of *Shh*, a key ligand in hedgehog signaling that promotes hepatocyte ballooning degeneration was induced by miR-34a and pro-fibrogenic cytokines *Tgfβ1* and *Tgfβ2* were induced at both mRNA and protein levels (Fig. 2B–D). Interestingly, treating AML12 cells with *Tgfβ2* enhanced the expression of miR-34a, suggesting a feedback regulation on the miR-34a-induced fibrogenic status (Fig. 2E). Collectively, these data demonstrated that miR-34a overexpression was sufficient to induce hepatocyte ballooning and a profibrogenic status in hepatocytes.

### miR-34a secreted from damaged hepatocytes mediated pathological communication between hepatocytes and HSCs

**Figure 3:**
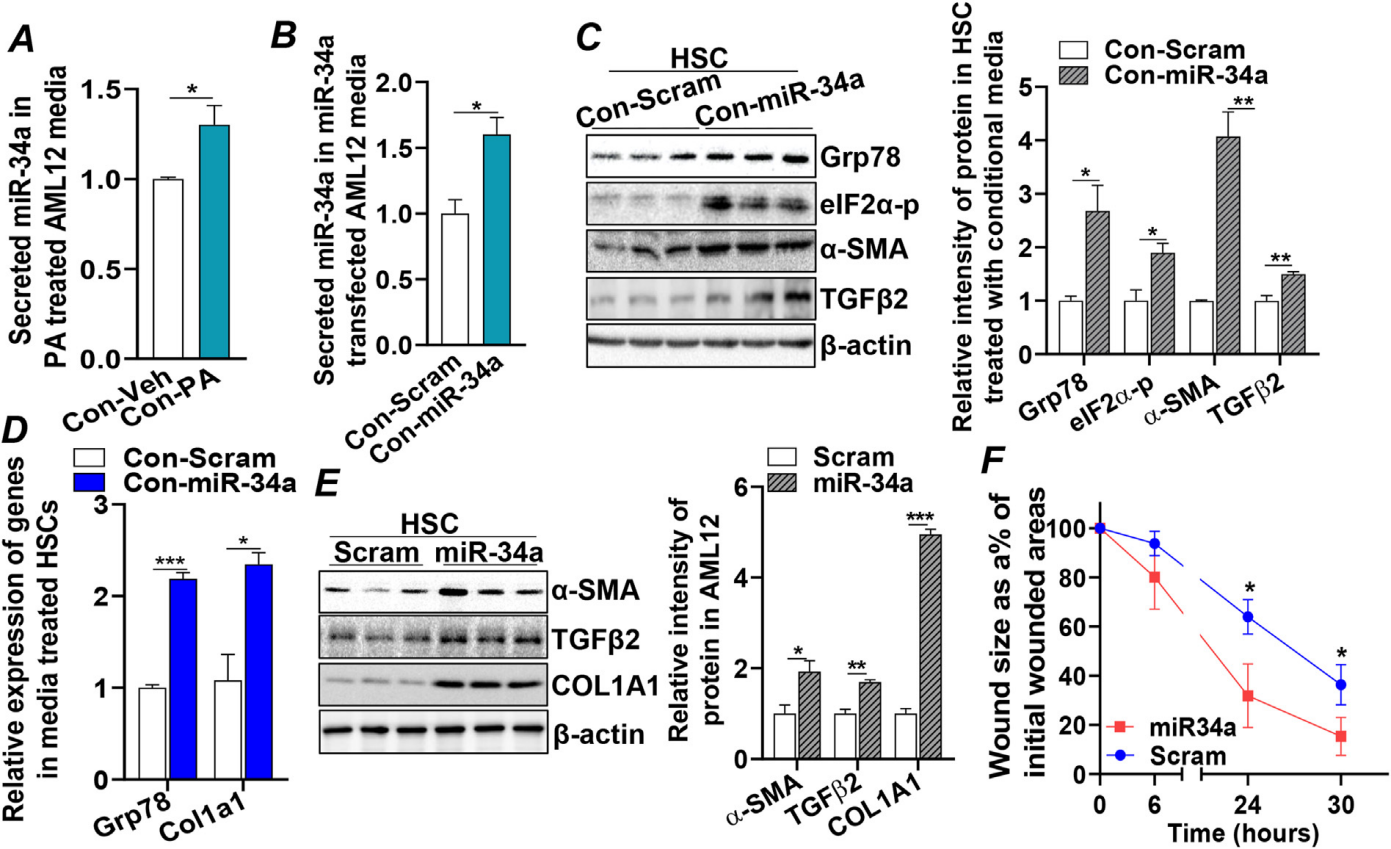
miR-34a secreted from damaged hepatocytes mediated pathological communication between hepatocytes and HSCs. **(A)** qRT-PCR analysis of miR-34a contents in the conditional media of AML12 treated with Veh (bovine serum albumin) or PA (0.25 mM) for 18 h. **(B)** qRT-PCR analysis of miR-34a contents in the conditional media of AML12 transfected with Scram or miR-34a mimic for 48 h. **(C, D)** Conditional media from AML12 transfected with Scram or miR-34a for 48 h were used to treat HSCs for 48 h, followed by (C) immunoblotting analysis of GRP78, α-SMA, elF2α-p, TGFβ2, and loading control β-actin proteins in the treated HSCs. (D) qRT-PCR of *Col1a1* and *Grp7*8 mRNAs in the treated HSCs. **(E)** Immunoblotting analysis of α-SMA, TGFβ2, COL1A1, and loading control β-actin protein in the HSCs transfected with Scram or miR-34a for 48 h. **(F)** Conditional media from AML12 transfected with Scram or miR-34a for 48 h were used to treat wounded monolayer HSCs. Images were captured at 0-, 6-, 24-, and 30-h timepoints. The rate of wound healing is presented by the ratio (%) of wounded areas at each indicated time point to its initial wounded area at the 0-h timepoint. Results represent mean ± standard deviation. The two-tailed student's *t*-test was used for statistical analyses of two-group comparisons. ∗*p* < 0.05, ∗∗*p* < 0.01, and ∗∗∗*p* < 0.001 versus controls. HSCs, hepatic stellate cells; qRT-PCR, real-time quantitative reverse transcription PCR; GRP78, glucose regulatory protein 78; eIF2α-p, phosphor-eukaryotic initiation factor-2α; JNK-p, phosphor-c-Jun N-terminal kinase; TGF-β2, transforming growth factor-beta 2; Veh, vehicle; Scram, scramble miRNA; PA, palmitic acid; α-SMA, alpha-smooth muscle actin; COL1A1, collagen type I alpha 1 chain.

Quiescent HSC activation is the key step to initiate hepatic fibrogenesis. To investigate if hepatocyte-derived miR-34a could induce HSC activation, we extracted miR-34a from the conditional media of PA-treated or miR-34a-transfected AML12 and found that higher amounts of miR-34a were present in the media of PA-treated or miR-34a-transfected cells compared with those in the mock-treated cells (Fig. 3A, B). Treating fresh hepatocytes (AML12 cells) with the conditional media collected from miR-34a-transfected cells (Con-miR-34a) activated ER stress markers GRP78 and eIF2α-p and induced expression of fibrogenic cytokine *Tgfβ2*, suggesting that the secreted miR-34a could act as a paracrine factor to impact the adjacent healthy hepatocytes (Fig. S2A). More importantly, incubation of HSC-T6 (HSC), rat HSCs, with Con-miR-34a also induced ER stress and fibrogenic status in the HSCs, indicated by the increased GRP78 and eIF2α-p, as well as the elevated expression of fibrogenic mediators, α-SMA, TGFβ2, and collagen type I alpha 1 chain (COL1A1) in treated HSCs (Fig. 3C, D). In untreated HSCs, trace levels of miR-34a were detected, which were much lower (∼10-fold) than those in the untreated AML12 hepatocytes (Fig. S2B). Incubation with Con-miR-34a significantly up-regulated miR-34a in HSCs and hepatocytes (Fig. S2B). To investigate the direct impact of miR-34a on HSCs, HSCs were transfected with miR-34a mimics. We found that miR-34a induced protein expression of α-SMA, TGFβ2, and COL1A1, a phenotype similar to what was observed in HSCs treated with con-miR-34a (Fig. 3C, D). This data further confirmed the deleterious effect of secreted miR-34a from injured hepatocytes on nearby hepatocytes and HSCs.

Next, a scratch-wound assay was implemented to test the effect of secreted miR-34a from hepatocytes on HSCs. We found that treating the scratch-wounded monolayer of HSCs with con-miR-34a significantly enhanced trans-differentiation and migration rate of HSCs, resulting in faster migration of HSCs into the cell-free gap, evidenced by the significantly smaller cell-free areas at 24 h and 30 h post-scratch compared with those treated with mock-conditional media (Fig. 3F; Fig. S2C, D). Together, these data demonstrate that miR-34a released from injured hepatocytes can mediate the deleterious impact to surrounding hepatocytes and HSCs, activating HSCs in a paracrine manner and inducing expression of fibrogenic mediators, *i.e.*, α-SMA, TGFβ2, and COL1A1, to sustain the development of liver fibrosis.

### Increased miR-34a in the livers of patients with steatosis, MASH, and liver fibrosis

**Figure 4:**
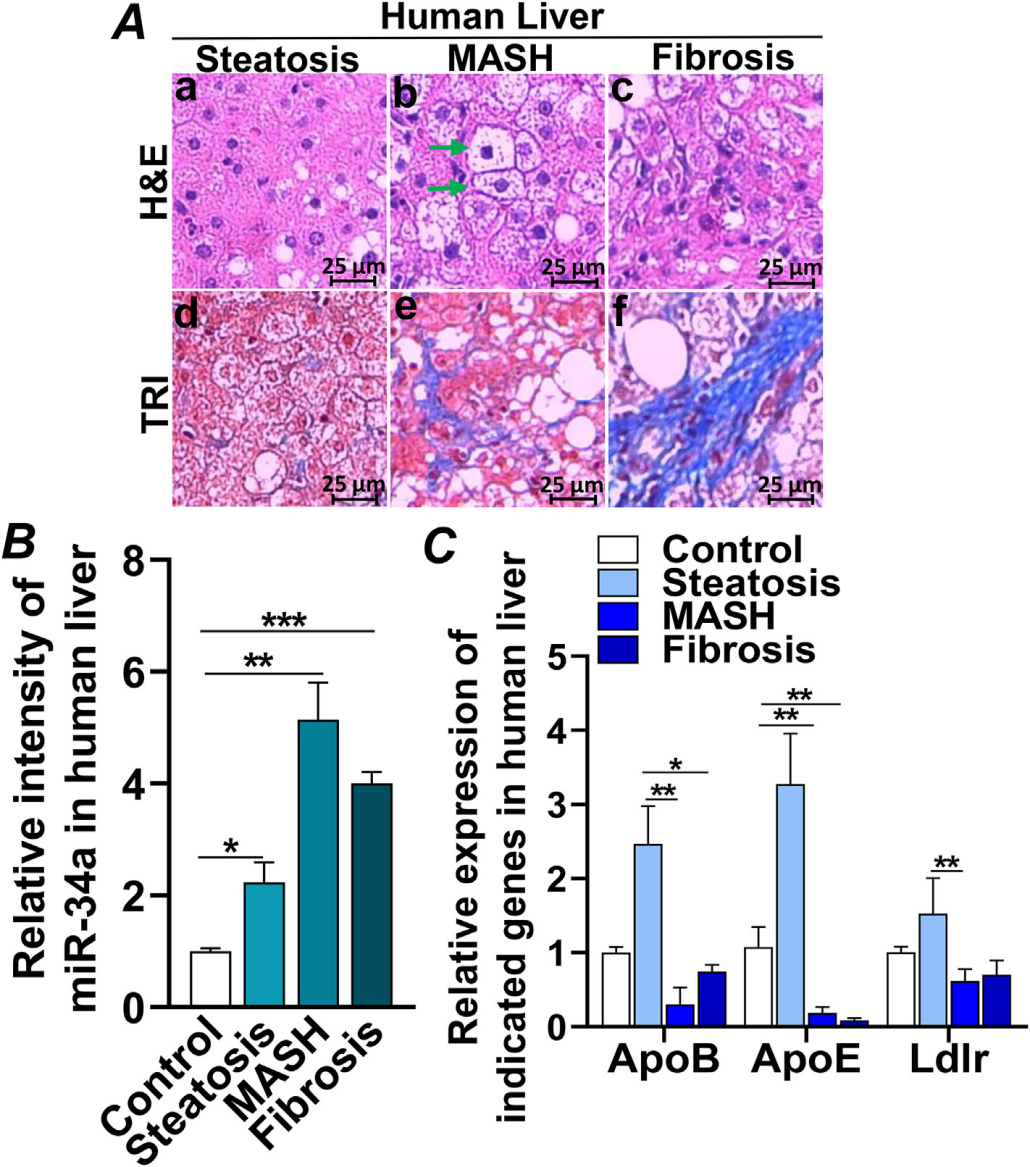
Increased miR-34a in the livers of patients with steatosis, MASH, and liver fibrosis. **(A)** Representative histological images of liver tissues from patients with steatosis, MASH, and liver fibrosis by hematoxylin–eosin or Masson's trichrome staining. The arrows mark the ballooned hepatocytes, and the circle mark the inflammatory monocyte infiltration. Scale bar, 25 μm. **(B, C)** Quantitative reverse transcription PCR analysis of mRNA expression of *ApoB*, *ApoE*, *Ldlr*, and miR-34a in the livers of controls and patients with steatosis, MASH, and liver fibrosis. Control, *n* = 3; steatosis, *n* = 7; MASH, *n* = 9; liver fibrosis, *n* = 9. Results represent mean ± standard deviation. The two-tailed student's *t*-test was used for statistical analyses of two-group comparisons. ∗*p* < 0.05, ∗∗*p* < 0.01, and ∗∗∗*p* < 0.001 versus controls. ApoB, apolipoprotein B; ApoE, apolipoprotein E; MASH, metabolic dysfunction-associated steatohepatitis; Ldlr, low-density lipoprotein receptor.

To investigate if miR-34a played a role in the development of MASLD, MASH, and liver fibrosis, liver tissues from obese patients were subjected to analysis by hematoxylin–eosin and Masson's trichrome staining. Fatty liver with no visual inflammatory foci and collagen deposition were found at steatosis (Fig. 4A–a & d; Fig. S3, a & d) whereas fatty liver with marked inflammatory monocyte infiltration, ballooned hepatocytes, and moderate collagen deposition were displayed at MASH (Fig. 4A, b & e; Fig. S3, b & e). Significant inflammation and broad fibrocollagenous tissue were developed in liver fibrosis (Fig. 4A–c & f; Fig. S3, c & f). Consistent with the histological changes, miR-34a was up-regulated by ∼2-fold at steatosis, which further increased and peaked at MASH (∼5-fold increase) and remained at the higher level (∼4-fold increase) when the liver developed fibrosis (Fig. 4B). Characterization of genes involved in hepatic lipid and lipoprotein metabolism uncovered a bi-phase pattern: *ApoB*, *ApoE*, and low-density lipoprotein receptor (*Ldlr*) were up-regulated at the early stage of MASLD (steatosis), which may be a compensatory response of the liver to cope with the overwhelming lipids accumulated in hepatocytes (Fig. 4C). These genes were markedly decreased at the stages of MASH and fibrosis (Fig. 4C), suggesting a decompensation of hepatic function when liver progressed to the advance stages of MASH and fibrosis. Together, these data demonstrated that miR-34a was significantly up-regulated in hepatic steatosis (MASLD), MASH, and liver fibrosis, which targeted the key metabolic pathways in the liver and resulted in liver dysfunction.

### Recapitulation of pathological conditions of patients with MASLD and liver fibrosis in a human MASLD-like mouse model

**Figure 5:**
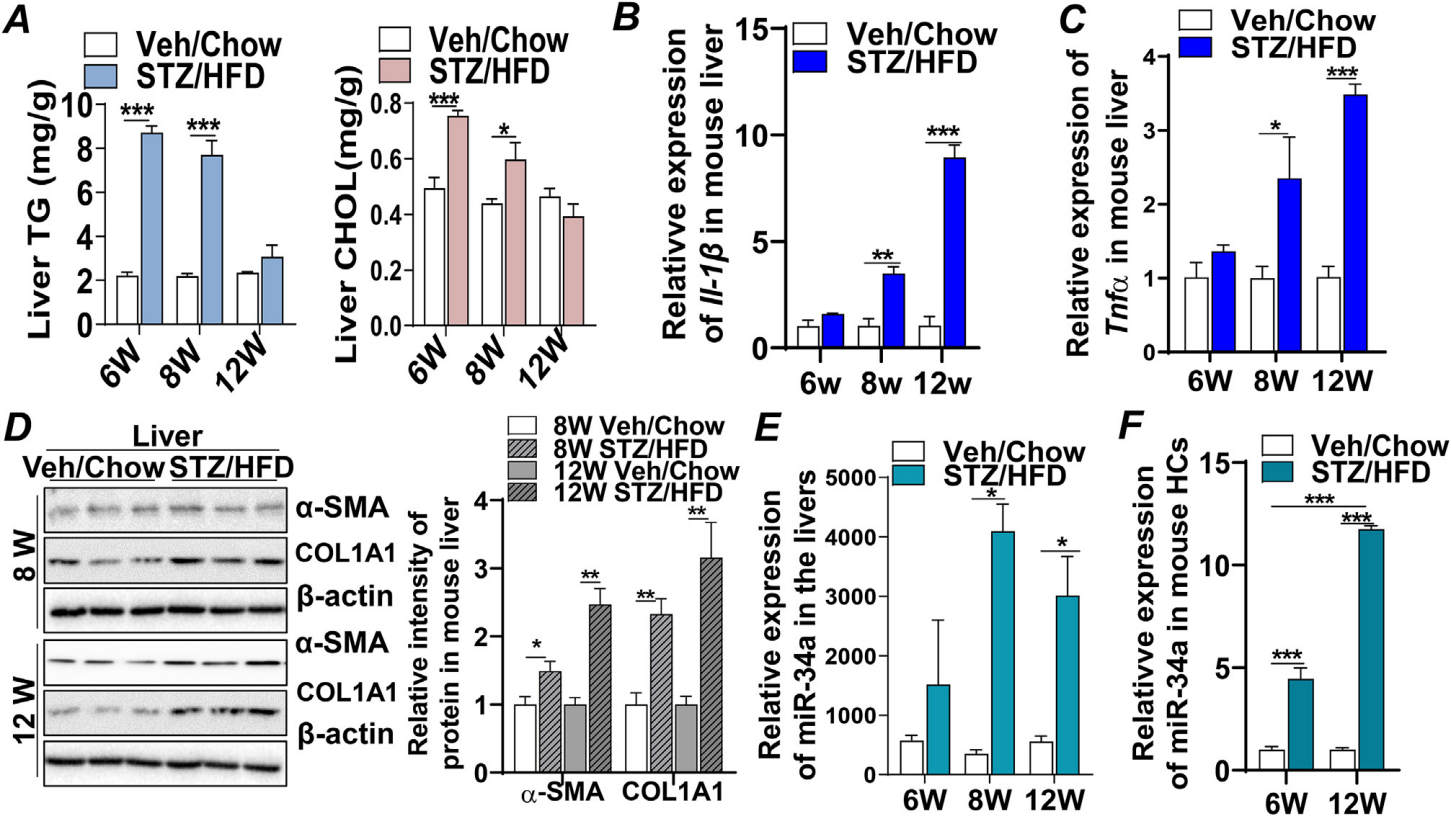
Recapitulation of pathological conditions of patients with MASLD and liver fibrosis in a human MASLD-like mouse model. Liver tissues from chow-fed controls or STAM mice (detailed in *Materials and methods*) at 6-, 8-, and 12-week-old were subjected to the following analysis. **(A)** TG and CHOL contents. **(B, C)** mRNA expression of *Il-1β* and *Tnfα.***(D)** Immunoblotting analysis of α-SMA, COL1A1, and loading control β-actin proteins. **(E)** miRNA deep sequencing counts of miR-34a. **(F)** mRNA expression of miR-34a in primary hepatocytes (HCs) of control or STAM mice at 6- and 12-week-old. *n* = 5–10/group. Results represent mean ± standard deviation. The two-tailed student's *t*-test was used for statistical analyses of two-group comparisons. ∗*p* < 0.05, ∗∗*p* < 0.01, and ∗∗∗*p* < 0.001 versus controls. MASLD, metabolic dysfunction-associated steatotic liver disease; TNFα, tumor necrosis factor alpha; Il-1β, interleukin-1β; α-SMA, alpha-smooth muscle actin; COL1A1, collagen type I alpha 1 chain; TG, triglyceride; CHOL, cholesterol.

To further investigate the molecular mechanism by which miR-34a drove progressive liver injury *in vivo*, a MASLD-like STAM mouse model was employed (detailed procedures in the *Materials and methods*) (Fig. S4A). Hyperglycemia was detected in STAM mice compared with that of controls (Fig. S4B). Analysis of mouse liver tissues collected at 6-, 8-, and 12-week-old mice by hematoxylin–eosin and Sirius Red staining demonstrated the development of a spectrum of pathological hallmarks similar to what was observed in patients with MASLD and liver fibrosis. Specifically, hepatic triglycerides and cholesterols contents were increased by approximately 4-fold or 1.5-fold in the liver tissues of STAM mice at 6 weeks old, which was accompanied by the accumulation of lipid droplets but no visual inflammatory foci and collagen deposition, indicating the development of simple steatosis (Fig. 5A; Fig. S4C, c & d). Increased mRNA expression of inflammatory cytokines *Il-1β* and *Tnfα*, infiltration of immune cells, and ballooning degeneration of hepatocytes were detected in the livers of 8-week-old STAM mice, suggesting the development of MASH (Fig. 5B, C; Fig. S4C, g & h). Activation of HSCs and fibrogenic signaling, such as increased expression of αSMA and COL1A1 proteins and collagen secretion, were observed in the livers of 8- and 12-week-old STAM mice while pro-fibrogenic cytokines *Tgfβ1/β2* were robustly induced at 12 weeks, demonstrating the presence of liver fibrosis (Fig. 5D; Fig. S4C, k & l; Fig. S4D, E).

More importantly, miRNA deep sequencing analysis of total miRNAs extracted from the STAM mouse livers and their age-matched controls revealed that miR-34a was ∼10-fold and ∼5-fold increased at 8 weeks and 12 weeks, respectively, compared with those in their chow-fed controls (Fig. 5E). These were the timepoints when the STAM mice developed MASH and fibrosis, consisting with the changes of miR-34a in the livers of patients with MASH and liver fibrosis (Figure 4, Figure 5). Alteration of miR-34a detected by miRNA sequencing was further verified by quantitative PCR analysis (Fig. S4F), supporting that miR-34a was up-regulated in the livers of mice with MASH and fibrosis. To pinpoint whether the increased miR-34a originated from hepatocytes, the expression of miR-34a was determined in primary hepatocytes isolated from the 6- and 12-week-old STAM mouse livers and found that miR-34a in STAM mouse hepatocytes was about 4-fold higher at steatosis (6 weeks) and 11-fold higher at fibrosis (12 weeks), compared with their age-matched chow-fed controls (Fig. 5F). Collectively, these data strongly demonstrated that the marked elevation of miR-34a was the key determinant that prompted liver injury from steatosis to MASH and fibrosis in a pathological manner that recapitulated the different disease stages of patients with steatosis, MASH, and liver fibrosis.

## Discussion

**Figure 6:**
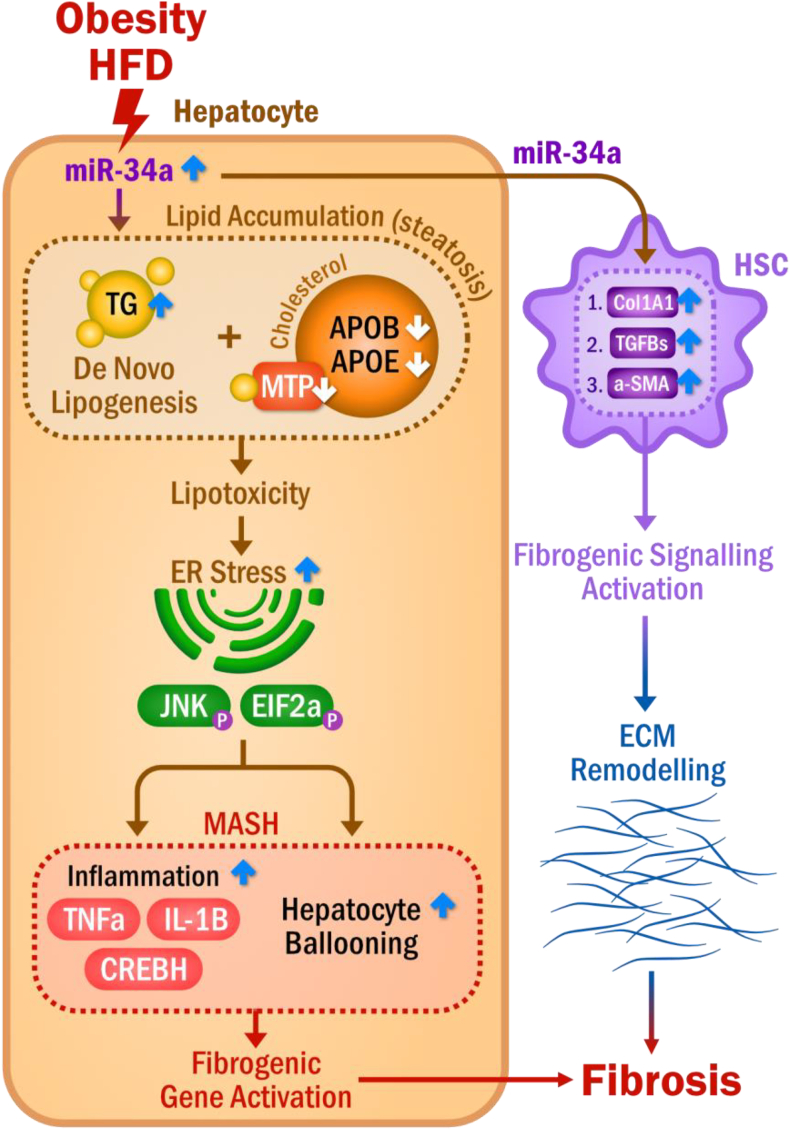
Increased miR-34a mediates pathophysiological communication between hepatocytes and HSCs in liver fibrosis. Increased miR-34a induced by obesity and high-fat diet enhances hepatic *de novo* lipogenesis and disturbs biosynthesis of very-low density lipoprotein metabolism via down-regulation of *Mttp*, *ApoB*, and *ApoE*, leading to the accumulation of lipids in the liver (steatosis). Lipotoxicity derived from the accumulated lipids further induces ER stress and activates metabolic inflammation in the liver, driving the progressive liver injury to MASH, characterized by the activation of TNFα, IL-1β, and CREBH, hepatocyte ballooning, and fibrogenic genes. Simultaneously, secreted miR-34a from the damaged hepatocytes contributes to HSC activation and the subsequent induction of fibrogenic signaling molecules, such as α-SMA, Col1A1, and TGF-β, promoting ECM remodeling and the development of liver fibrosis. TNFα, tumor necrosis factor-alpha; ApoB, apolipoprotein B; ApoE, apolipoprotein E; Mttp, microsomal triglyceride transfer protein; Il-1β, interleukin-1β; CREBH, cAMP-responsive element binding protein H; TGF-β, transforming growth factor-beta; HSC, hepatic stellate cell; COL1A1, collagen type I alpha 1 chain; MASH, metabolic dysfunction-associated steatohepatitis; α-SMA, alpha-smooth muscle actin; ER, endoplasmic reticulum; ECM, extracellular matrix.

Research in this study presents three novel mechanisms of miR-34a: i) miR-34a overexpression induced by lipotoxicity can activate ER stress and hepatocyte ballooning degeneration (Fig. 6). ii) Secreted miR-34a from injured hepatocytes mediates pathophysiological communication between hepatocytes and HSCs, which can activate fibrogenic signaling and enhance liver fibrosis (Fig. 6). iii) This study, for the first time, systemically characterizes the adverse impact of increased miR-34a in the progressive liver injury from MASLD, MASH, and liver fibrosis in both human patients and in a human MASLD-like mouse model.

The global prevalence of MASLD represents an alarming health crisis that is expected to be the leading cause of liver transplantation.^30^^,^^31^ However, there are currently no standard diagnostic markers and effective treatments,^32^ imposing an urgent need to conduct research that can delineate the underlying mechanism and lead to the identification of therapeutic targets to delay, halt, or reverse liver fibrosis. miR-34a was reported to be involved in MASLD by targeting peroxisome proliferator-activated receptor-α (PPARα) expression and lipoprotein metabolism.^33^^,^^34^ In this study, we investigated the novel mechanism of increased miR-34a induced by lipotoxicity in progressive liver injury from steatosis to MASH and fibrosis. Up-regulation of miR-34a was associated with all the pathological hallmarks of chronic liver disease, including activation of metabolic inflammation, hepatocyte ballooning degeneration, pro-fibrogenic signaling, and extracellular matrix production in clinical patients, in the liver of a human MASLD-like mouse model, and hepatocytes. We further demonstrated that secreted miR-34a from damaged hepatocytes mediated the pathological communication between hepatocytes and HSCs, leading to the activation of fibrogenic signaling in HSCs and the remodeling of the extracellular matrix that contributed to liver fibrosis.

Hepatocyte death is a crucial initial event in all liver diseases. Danger-associated molecular pattern signaling molecules released from damaged hepatocytes in fatty liver disease confer danger signals to surrounding cells, including HSCs, Kupffer cells, and the nearby hepatocytes, which drive further liver injury and, therefore, play a significant role in fibrosis development.^35^, ^36^, ^37^ In this study, we found that an increase expression of miR-34a in hepatocytes enhanced the expression of pro-inflammatory cytokines, TNFα and IL-1β, contributing to inflammatory activation in the damaged hepatocytes. Moreover, secreted miR-34a from damaged hepatocytes may further induce inflammatory responses in adjacent healthy hepatocytes via the mediation of TLR4 and CREBH. The passive release of miR-34a from damaged hepatocytes further exerts a biological impact on HSCs as incubation of HSCs with the conditional media collected from miR-34a-transfected AML12 induced ER stress and activated profibrogenic status in the treated HSCs. These phenotypes were recapitulated in HSCs directly transfected with miR-34a mimics, suggesting the key role of secreted miR-34a in mediating communication between injured hepatocytes and HSCs and their subsequent activation. The enhancement of wound healing in the scratch-wounded assay of HSCs further verified the secreted miR-34a on HSC activation and migration. The activated HSCs, as the main executors of liver fibrosis, interact with liver-resident cells and produce extracellular matrices, leading to liver fibrosis. miRNA-mediated cell–cell communication has also been reported in cancer metastasis and other diseases. For example, exosome-mediated transfer of miR-105 from metastatic breast cancer cells to endothelial cells through targeting a tight junction protein, zonula occludens 1 (ZO-1), destroyed the endothelial barrier function and promoted metastasis.^38^^,^^39^ miR-21-3p in the exosomes from umbilical cord blood was also found to promote the proliferation and migration of fibroblasts, which induced the angiogenic activities of endothelial cells and accelerated wound healing.^40^ Thus, secreted miRNAs may act as a chemical messenger to mediate cell–cell communications among damaged hepatocytes, nearby healthy hepatocytes, and HSCs, contributing to the progression of MASLD towards fibrosis.

One of the most challenging steps in the study of MASLD is to establish an animal model that can recapitulate the pathological progression path in human liver disease. Several chemicals or diet-induced liver fibrosis mouse models have been utilized in biomedical research. CCl_4_-induced liver fibrosis is one of these mouse models. However, liver injury induced by CCl_4_ is the consequence of chemical toxicity, which does not resemble the pathological path of MASLD and liver fibrosis in humans. The dietary methionine choline deficient (MCD) mouse model is another frequently utilized animal model for MASH and liver fibrosis research.^41^, ^42^, ^43^ The MCD diet in this model usually consists of sucrose and fat but is devoid of methionine and choline, which causes impairment on hepatic fatty acid β-oxidation and decrease of VLDL synthesis.^44^ Therefore, in addition to prominent steatosis and extensive necroinflammation, the MCD-fed mice also show significant weight loss, no insulin resistance, and low serum insulin, fasting glucose, leptin, and triglyceride levels,^45^ a metabolic profile opposite to what has been observed in obese patients with MASLD. The STAM model presented in the current study is an animal model of MASLD in which the pathological hallmarks of steatosis, MASH, and liver fibrosis, such as hepatic lipid accumulation, metabolic inflammation, and hepatocyte ballooning degeneration, and fibrogenesis, were developed in a predictable time-scale, and more closely recapitulated the pathological phenotypes in human patients at the different disease stage of MASLD and fibrosis.^46^ The STAM model would provide a novel platform to delineate the intricate mechanisms linking metabolic disorder and liver fibrosis with a goal to develop novel therapeutic strategies against MASLD and liver fibrosis.

## Conclusions

This study, for the first time, uncovered a novel mechanism of miR-34a in the activation of hepatic *de novo* lipogenesis, metabolic inflammation, hepatocyte ballooning degeneration, and liver fibrogenic signaling induced by lipotoxicity. Induction of these pathological hallmarks contributed to progressive liver injury in steatosis, MASH, and liver fibrosis. Novel findings from this study may lend support to the development of a pharmaceutical strategy that can manipulate the expression of miR-34a in MASLD and halt the disease progression toward liver fibrosis.

## CRediT authorship contribution statement

**Qihua Duan:** Writing – review & editing, Writing – original draft, Software, Methodology, Investigation, Formal analysis, Data curation. **Ruixiang Hu:** Writing – review & editing, Data curation. **Yan Chen:** Writing – review & editing, Investigation. **Henry Wade:** Writing – review & editing, Investigation. **Szczepan Kaluzny:** Writing – review & editing, Investigation. **Bingrui Zhang:** Investigation, Validation, Writing – review & editing. **Rongxue Wu:** Conceptualization, Writing – review & editing. **Guangnan Liu:** Supervision, Writing – review & editing. **Cunchuan Wang:** Data curation, Writing – review & editing. **Edward N. Harris:** Writing – review & editing, Supervision, Funding acquisition, Conceptualization. **Qiaozhu Su:** Writing – review & editing, Writing – original draft, Supervision, Funding acquisition, Conceptualization.

## Data availability

The datasets supporting the conclusions of this article are available from the corresponding author upon reasonable request.

## Funding

This work was supported by the British Heart Foundation (UK) (No. PG/19/86/34,788 to Q.S.), Northern Ireland Chest Heart & Stroke (UK) (2019_08 to Q.S.), NIH grant R01 GM147913 (to E.N.H.), and a Nebraska Research Initiative grant (to E.N.H.).

## Conflict of interests

The authors declared no conflicts of interest with the contents of this article.

## Footnotes

Appendix A Supplementary data to this article can be found online at https://doi.org/10.1016/j.gendis.2025.101648.
