# Supplementary material for: Elevated miR-34a induced by lipotoxicity and inflammation mediates pathophysiological communication between hepatocytes and hepatic stellate cells in liver fibrosis
Source: Genes Dis. 2025 Apr 18;12(6):101648. doi: 10.1016/j.gendis.2025.101648 (PMC12361996; doi:10.1016/j.gendis.2025.101648)
Supplement: Multimedia component 1 [file mmc1.pdf]

Supplementary Materials

Elevated miR-34a induced by lipotoxicity and inflammation mediates pathophysiological communication between hepatocytes and hepatic stellate cells in liver fibrosis

Qihua Duan[1], Ruixiang Hu[2], Yan Chen[3], Henry Wade[1], Szczepan Kaluzny[1], Bingrui Zhang[1], Rongxue Wu[4], Guangnan Liu[3], Cunchuan Wang[2], Edward N. Harris[5], Qiaozhu Su[1]

[1]Institute for Global Food Security, School of Biological Sciences, Queen's University Belfast, Belfast, BT9 5DL, United Kingdom

[2]Department of Gastrointestinal Surgery, First Affiliated Hospital of Jinan University, Guangzhou, China

[3]Second Affiliated Hospital of Guangxi Medical University, Guangxi, China

[4]Department of Medicine, Section of Cardiology, University of Chicago, Chicago, USA

[5]Department of Biochemistry, University of Nebraska-Lincoln, Lincoln, NE, 68588, USA

Running Title: miRNA-34a in MASLD, MASH and liver fibrosis

To whom materials & correspondence should be addressed:

Dr. Qiaozhu Su, MD, PhD

Associate Professor

Institute for Global Food Security

School of Biological Sciences

Queen's University Belfast,

Belfast, United Kingdom

BT9 5AG

Tel: +44 (0)28 90 97 4388

Email: q.su@qub.ac.uk

Supplementary Materials and Methods

Cell culture, transfection, and treatments

A mouse liver hepatocyte cell line (AML12) and rat liver stellate cell line (HSC-T6) were obtained from America Type Culture Collection (ATCC). AML12 cells were cultured with DMEM/F-12 medium (Gibco) supplemented with 10% FBS (Gibco), 1% insulin, transferrin, selenium (ITS; Gibco), 1% Penicillin Streptomycin (PS) (Gibco), and 40 ng/mL dexamethasone. HSC-T6 was maintained in Dulbecco's Modified Eagle Medium (DMEM) containing 10% FBS. Cells were grown in T75 flasks at 37°C and 5% CO_2. For miRNA transfection studies, $3x10^5$ cells were seeded onto 6-well plates. The next day, cells reached 60~70% confluence and were transfected with scramble miRNA mimics (30nM, Qiagen, GeneGlobe ID - YM00479902) or miR-34a-5p mimics (30nM, Qiagen, GeneGlobe ID - YM00473212) using Lipofectamine 3000. 6-hours post-transfection, culture medium was replaced with fresh medium containing 10% FBS for additional 48-hours.

For palmitic acid (PA) treatment: A 20mM stock solution of PA (Sigma-Aldrich) was prepared in water at 60℃ and filtered before use. Filter-sterilized PA stock solution was added to DMEM containing 0.5% fatty acid free bovine serum albumin (BSA; Sigma-Aldrich) to obtain 0.25mM PA and incubated at 60℃ for 10 minutes. The PA-BSA conjugated DMEM media was added immediately to cells. (i) For AML12 cell treatments: AML12 cells were seeded onto 6-well plates ($1x10^6$). The next day, cells were treated with either 0.25mM PA or vehicle (PBS) for 18h. For PA plus tumour necrosis factor (TNF)α treatment, cells were treated with 0.25mM PA or vehicle (PBS) for 6-hours followed by TNFα (Thermo Fisher Scientific) at final concentation of 20 ng/ml for additional 12-hours. There were three replicates for each cell treatment, and the experiment was repeated 3-times. (ii) For HSC cell treatment: HSC cells were seeded onto 6-well plates ($1x10^6$). The next day, cells were treated with conditional media from AML12 for 48h.

Immunoblot analyses

Cells and mouse liver tissues were lysed with 1x radioimmunoprecipitation assay (RIPA) lysis buffer. Protein concentrations of the tissue and cell lysates were determined by the Bradford dye-binding method (Bio-Rad). Immunoblotting analysis was performed as previously described [2].The following antibodies were used in this study: anti-Grp78 (Cell Signalling, 3183S), anti-eIF2α-p (Cell Signalling; 3597S), anti-JNK-p (Cell Signalling; 9255S), anti-TLR4 (Cell signalling, 14358S), anti-Traf6 (cell signalling, #8028), anti-α-SMA (Santa cruz; SC-53015), anti-COL1A1 (Cell Signalling; 84336S), anti-CREBH(NOVUS, NBP2-16008), anti-TGFβ_1 (Santa cruz; SC-146), anti-TGFβ_2 (Abcam, ab36495), and β-actin (Proteintech, 81115-1-RR). Secondary antibodies included anti-Mouse (Cell

signalling; 7076S), anti-Rabbit (Cell Signalling, 7074S), anti-Goat (Abcam; 7076S). All antibodies were used at a final concentration of 0.1–1 µg/mL, signals were detected using enhanced chemiluminescence (Pierce, Rockford IL, USA).

RNA isolation, reverse transcription, and qRT-PCR

Total RNA was isolated from tissues or cells using TRIzol (Life Technologies). RNA integrity was confirmed using a NanoDrop 2000 (Wilmington, DE). First strand cDNA was synthesized with oligo (dT), random primers and specific miRNA primers using a high-capacity cDNA reverse Transcription kit with RNase inhibitor (Life Technologies). Q-RT-PCR reactions were performed on Roche LighterCycler 480 using SYBR Green PCR master mix (Applied Biosystems; Life Technologies). Relative induction of gene mRNA and miRNA expression was calculated using the expression of 18s rRNA and sno202, respectively, for normalization.

Lipid extraction

From cell lysate: to 1 mL of whole cell lysate, 2 mL Folch/BHT reagent (2:1 chloroform:methanol + 100 µg/mL BHT) was added. Solutions were mixed by vortex and centrifuged at 2000 xg for 10-mins. After centrifuge, the lower clear phase of solution was transferred to a clean glass tube. The solvents were evaporated under N2 gas using a Turbovap. The remaining lipid solution was dissolved in 1 mL chloroform + 1% triton x-100 and dried again using the Turbovap. The remaining lipid/triton solution was dissolved in 300 µL of water.

miRNA fluorescent in situ hybridization (FISH)

RNA probe was transcribed using Biotin RNA Labelling Mix (Roche; 11685597910) and T7 RNA polymerase (10881767001). AML12 cells were seeded onto coverslips. After 24-hours, cells were fixed in 10% neutral buffered formalin before permeabilization using 0.1% triton x-100. After washing, cells were then dehydrated in an ethanol series using 70%, 80%, then 95% ethanol for a period of 2-mins each and air-dried. Cells were submerged in 1 mL hybridisation solution (500 µL 2X SSC buffer + 500 µL 8M urea, 100 mg DSS + 40 nmol/L biotin-labelled miR-34a probe +10 µL RNAse inhibitor) and heated to 80°C for 2-min, the temperature was decreased to 55°C for 2-hours, then reduced to 37°C and allowed to incubate overnight. Cells were washed then incubated in DEPC-treated PBS + Hoechst 33342 + streptavidin FITC (Biolegend; 405201) for 30 mins at 37°C in dark. Microscope images were captured on a Stellaris 5 confocal microscope.

Supplementary Figures

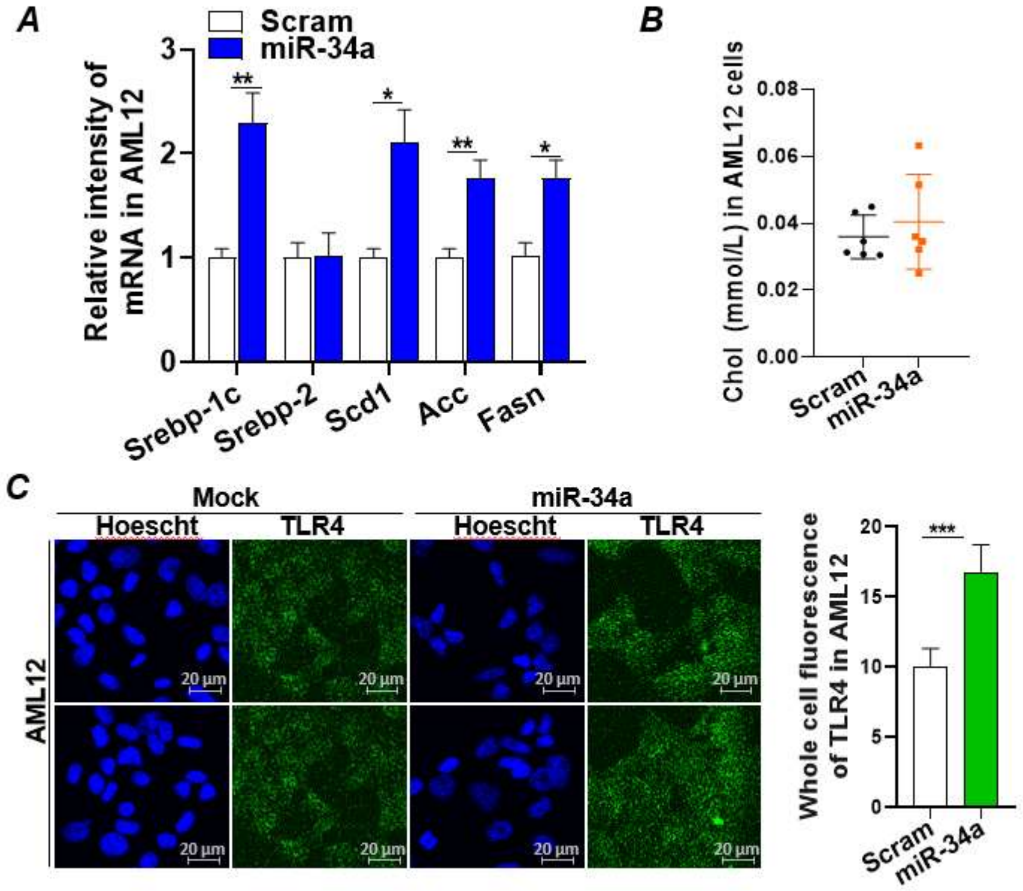

Supplementary Figure 1. (A) Relative mRNA expression of *Srebp-1*, *Srebp-2*, *Scd1*, *Acc,* and *Fasn* by qRT-PCR in AML12 cells transfected with Scramble (Control) or miR-34a mimics for 48-hours. **(B)** CHOL contents in the AML12 transfected with Scramble (Control) or miR-34a mimics for 48-hours. **(C)** Immunofluorescent confocal images of AML12 cells transfected with Scramble or miR-34a mimics for 48-hours followed by immune-stained with anti-TLR4 antibody and Hoechst 33342. Scale bar represents 20μm. For cell treatment, two independent experiments were performed in triplicate. Results represent the mean ± SD. The two-tailed Student t test was used for statistical analyses of two-group comparisons. *P < 0.05, **P < 0.01 and ***P < 0.001 vs. controls.

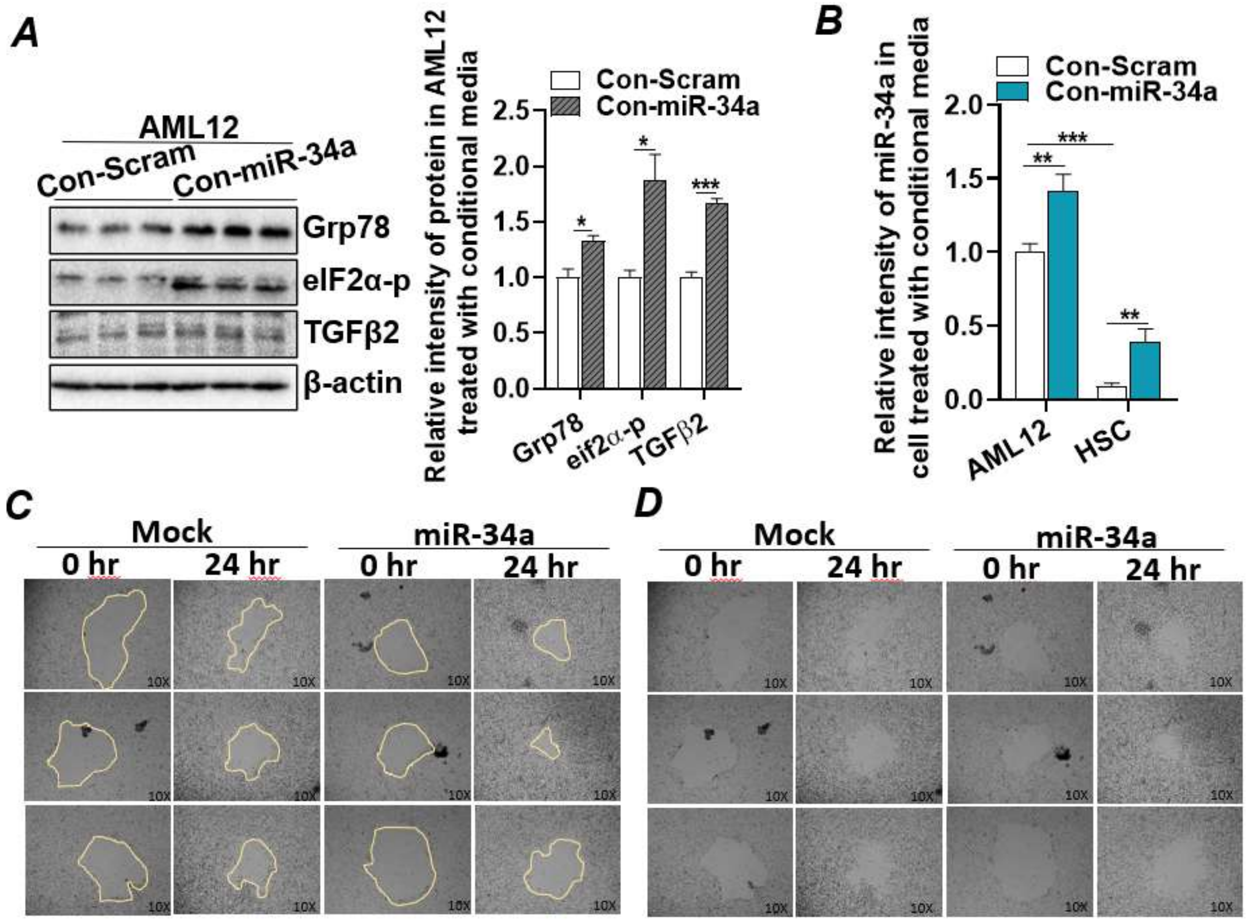

Supplementary Figure 2. (A) Conditional media from AML12 transfected with Scramble or miR-34a for 48-hours were used to treat AML cells for 48-hours. Immunoblotting analysis of GRP78, α-SMA, elF2α-p, TGFβ2 and loading control β-actin proteins in the treated AML12 cell lysates. **(B)** qRT-PCR of miR-34a mRNAs in AML12 cells and HSC cells treated with conditional media collected from AML12 cells transfected with Scramble or miR-34a for 48-hours. **(C-D)** Wound healing assay: Conditional media from AML12 transfected with Scramble or miR-34a for 48-hours were used to treat wounded monolayer HSC cells. Representative images of the wounded areas at 0 hour and 24 hours. Results represent the mean ± SD. The two-tailed Student t test was used for statistical analyses of two-group comparisons. *P < 0.05, **P < 0.01 and ***P < 0.001 vs. controls.

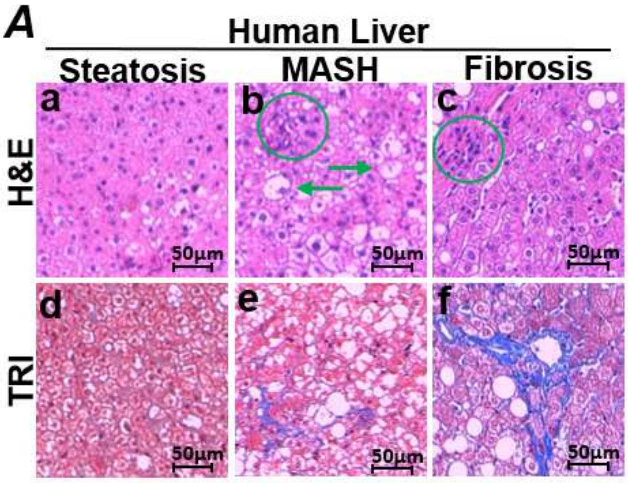

Supplementary Figure 3. (A) Representative histological images of liver tissues from patients with steatosis, MASH and fibrosis by H&E or Masson's Trichrome staining. Arrows marked the ballooned hepatocytes; Circle marked the inflammatory monocyte infiltration. Data are presented for the following groups: Control (n=3), Steatosis (n=7), NASH (n=9), and Liver Fibrosis (n=9).

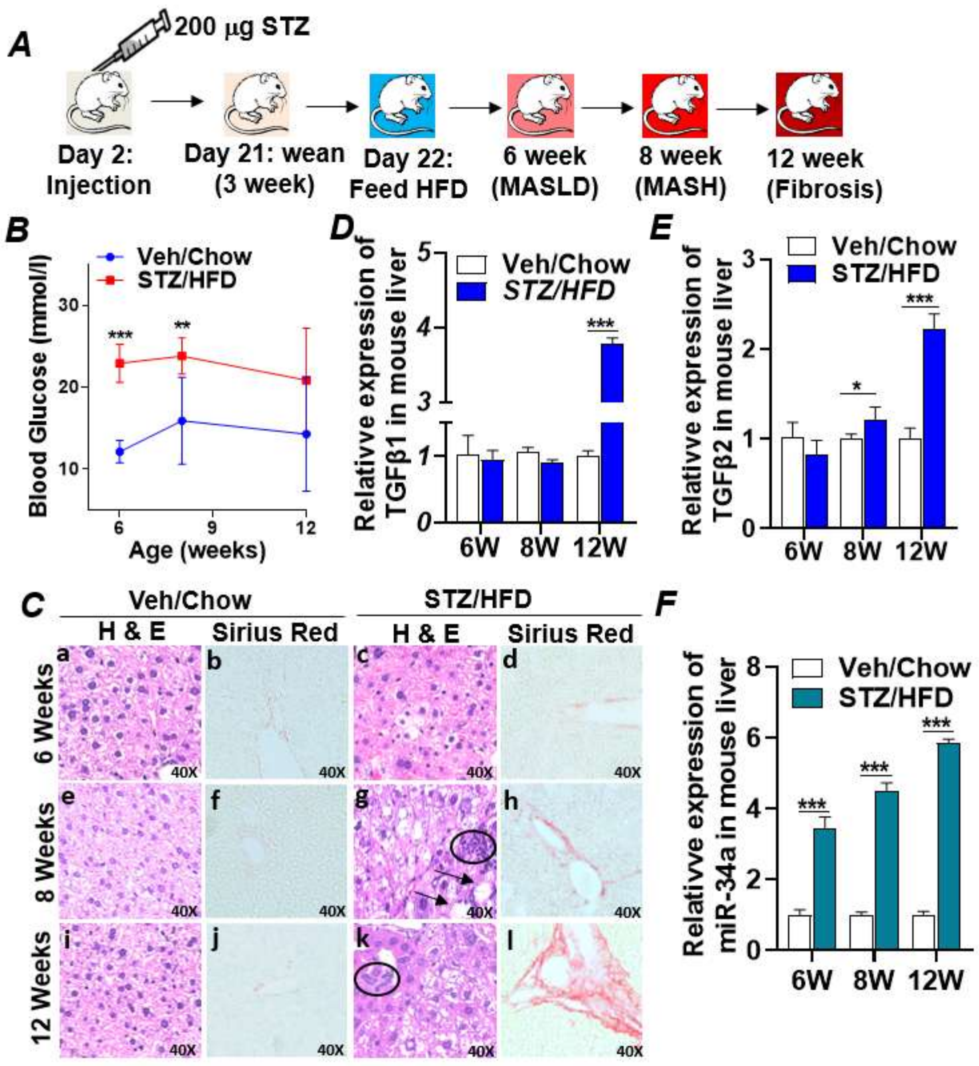

Supplementary Figure 4. (A) The indicated groups of male mice (C57BL/6J) mice were injected with Veh or STZ at 2-days old followed by feed with HFD from 22-days old for up to 12-weeks (n = 5–10/groups). Plasmas and liver tissues were collected for protein and RNA extractions followed by analysis. **(B)** Blood Glucose of control or STAM mice. (**C)** Representative histological images of liver tissues by H&E and Sirius Red staining. Arrows marked the ballooned hepatocytes; Circle marked the inflammatory monocyte infiltration. **(D-E).** qRT-PCR of mRNA expression of *Tgfβ1* and *Tgfβ2* in the livers of control or STAM mice at 6 weeks, 8 weeks and 12 weeks old. **(F)** Expression of miR-34a in the livers of control or STAM mice at 6-weeks, 8-weeks and 12-weeks old. Results represent the mean ± SD. The two-tailed Student t test was used for statistical analyses of two-group comparisons. *P < 0.05 and ***P < 0.001 vs. controls.

Sequences of primers used in this study

q-RT-PCR		
Mouse	Forward (5`-3`)	Reverse (5`-3`)
18s	TAAGTCCCTGCCCTTTGTACACA	GATCCGAGGGCCTCACTAAAC
ACC	ACAGTGGAGCTAGAATTGGAC	ACTTCCCGACCAAGGACTTTG
ApoB	TCACCAGTCATTTCTGCCTTTG	CGTGGGCTCCAGCATTCTA
ApoE	CACAATTGCGAAGATGAAGGC	TTTGCCACTCGAGCTGATC
COL1A1	TTCTCCTGGCAAAGACGGAC	CCATCGGTCATGCTCTCTCC
CREBH	GGCCATTGACCTGGACATGT	TTCACAGTGAGGTTGAAGCGG
Fasn	TGGGTTCTAGCCAGCAGAGT	ACCACCAGAGACCGTTATGC
Grp78	TGCATCTGATGGTTCAGCTC	GTGTGCATGCCATGTGTGTA
IL-1β	TGGGCTGGACTGTTTCTA	ATCAGAGGCAAGGAGGAA
IL-6	GTCCTTAGCCACTCCTTCTG	TTCCATCCAGTTGCCTTCT
MttP	TGGTGAAAGGGCTTATTCTGTT	TTGCAGCTGAATATCCTGAGAA
PDGFR β1 (FGFR)	TCATCCCCATTCCAAACTGTG	GCTTCACCCCTGTCTTCC
Scd	GAGAAGGGCGGAAAACTGGA	TGAAGCACATCAGCAGGAGG
SHH	CGAGACCCAACTCCGATGTG	ATATAACCTTGCCTGCCGCT
Srebp-1c	GAGGCCAGAGAAGCAGAAGAG	GAACAGACACTGGCCGAGAT
Srebp-2	TGGTAGGTCTCACCCAGGAG	CAGGGGTCTTCAGCATGATT
TGF-β1	AGCAACATGTGGAACTCTACC	GAAAGCCCTGTATTCCGTCTC
TGF-β2	CTACAGACCCTACTTCAGAATCG	CGTCGAAGGAGAGCCATTC
TNFα	GTCTCATTCCTGCTTGTGGC	GCACTTGGTGGTTTGCTACG
SNO202	AGTACTTTTGAACCCTTTTCCA	GTGCAGGGTCCGAGG
MiR-34a	GCTGGCAGTGTCTTAGCT	
	RT: GTCGTATCCAGTGCGTGTCGTGGAGTCGGCAATTGCACTGGATACGACACAACC	
Human	Forward (5`-3`)	Reverse (5`-3`)
ApoE	GGACGTCCTTCCCCAGGA	GGGGTCAGTTGTTCCTCCAG
IL-1β	GCTCTCCACCTCCAGGGACA	AGGCCCAAGGCCACAGGTAT
IL-6	GTAGTGAGGAACAAGCCAGAG	GAACTCCTTAAAGCTGCGC

LDLR	GGACGTCCTTCCCCAGGA	CACATTAACGCAGCCAACTTC
TNFα	CCTTCCTGATCGTGGCAG	GCTTGAGGGTTTGCTACAAC
